# Methimazole-Induced Pleural Effusion in the Setting of Graves' Disease

**DOI:** 10.1155/2019/5748938

**Published:** 2019-08-04

**Authors:** Le Yu Khine, Dong Won Kim, Omolola Olajide, Chelsey White, Yousef Shweihat, Henry Driscoll

**Affiliations:** ^1^Division of Endocrinology, Marshall University, Joan C. Edwards School of Medicine, USA; ^2^Division of Internal Medicine, University of Maryland School of Medicine, USA; ^3^Division of Pulmonary and Critical Care and Sleep Medicine, Marshall University, Joan C. Edwards School of Medicine, USA

## Abstract

Methimazole is a thionamide drug that inhibits the synthesis of thyroid hormones by blocking the oxidation of iodine in the thyroid gland. We report a case of methimazole-induced recurrent pleural effusion. A 67-year-old female with recently diagnosed Graves' disease on methimazole 20mg daily was admitted with dyspnea and new onset atrial fibrillation with rapid ventricular rate. Chest X-ray revealed a unilateral right pleural effusion, which was consistent with a transudate on thoracocentesis. She was managed as a case of congestive heart failure and methimazole dose was increased to 30 mg daily. She was readmitted twice with recurrent right pleural effusion. The fluid revealed an exudative process on repeat thoracocentesis. CT scan of the chest with contrast showed mediastinal lymphadenopathy and a diffuse ground glass process involving the right lower lobe suggestive of pneumonitis. Bronchoalveolar lavage showed neutrophil predominant fluid, and cytology and adenosine deaminase were negative. Patient also had an endobronchial ultrasound guided biopsy of the lymph nodes (EBUS). She was treated empirically with steroids 40 mg for 10 days and the methimazole was also discontinued. The antinuclear antibodies (ANA) came back positive with a speckled pattern; antineutrophil cytoplasmic antibody (c-ANCA) and antimyeloperoxidase were also positive. The effusion resolved but recurred on rechallenge with methimazole. She was referred for urgent thyroidectomy. The patient's repeat chest X-ray showed complete resolution of the pleural effusion after stopping the methimazole. Few weeks later, repeat ANCA and antimyeloperoxidase antibody were both negative. Our case report highlights the importance of the recognition of a rare side effect of methimazole. Timely diagnosis would ensure that appropriate treatment is given.

## 1. Introduction

Methimazole is a thionamide drug that inhibits the synthesis of thyroid hormones by blocking the oxidation of iodine in the thyroid gland. It has been associated with a significant number of side effects. ANCA-associated vasculitis is a well-known complication of propylthiouracil, another thionamide drug. Methimazole has been recently implicated in inducing ANCA vasculitis [1].

There are reported cases of methimazole-induced pulmonary hemorrhage; however, other complications such as pleural effusion are very rare with methimazole [2–4]. There have been two reported cases of methimazole associated pleural effusions [2, 3]. We report a case of methimazole-induced recurrent pleural effusion in a woman with Graves' Disease.

## 2. Case Presentation

We present a 67-year-old female admitted with dyspnea and new onset atrial fibrillation with rapid ventricular rate. She was diagnosed with Graves' disease 3 weeks earlier and had been started on 20 mg of methimazole daily. Thyroid function tests showed suppressed TSH with elevated Free T3 and Free T4. Details of lab results are given in Table 1. Pro-BNP was elevated at 1762. Chest X-ray revealed unilateral right pleural effusion which was subsequently drained by thoracentesis and was consistent with transudative effusion. She was managed as a case of congestive heart failure. Her heart rate control was optimized and the dose of methimazole was increased to 30 mg once daily. She was discharged home but readmitted 2 days later with recurrent dyspnea. A repeat chest X-ray showed recurrence of the right pleural effusion. Transthoracic echocardiogram was done showing normal ejection fraction and diastolic function. Her heart rate was controlled. Repeat thyroid function tests showed a normal free T3 and almost normal free T4. Patient was diuresed, and the unilateral right pleural effusion was drained again. The fluid characteristics showed a transformation from transudative to exudative likely in the setting of recent diuretic use.

**Figure 1:**
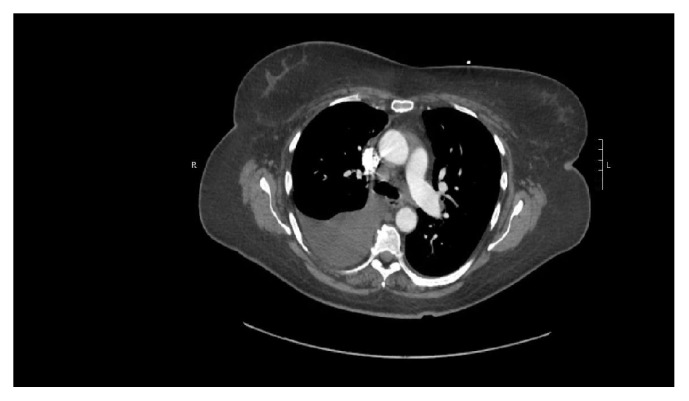
CT of chest showing mediastinal lymphadenopathy with presence of pleural effusion.

**Figure 2:**
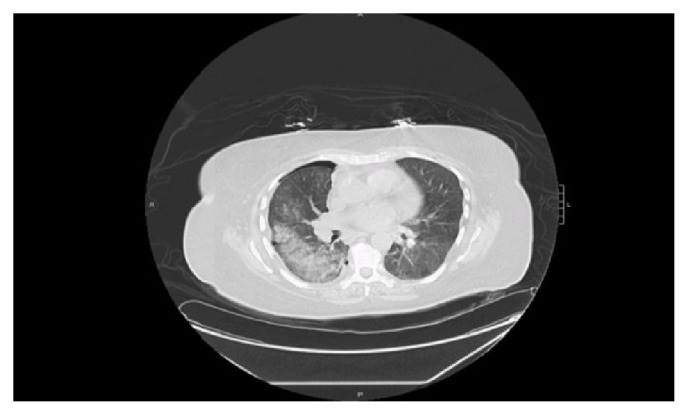
Presence of ground glass opacity suggesting pneumonitis after removal of pleural fluid.

She was then discharged home after she had improved clinically only to be readmitted again with worsening dyspnea attributed to reaccumulation of the right pleural fluid a few days later. A repeat drainage with pleural catheter was performed, and the fluids revealed an exudative process with elevated LDH. This was further evaluated with a CT scan of the chest with contrast (Figures 1 and 2) which showed mediastinal lymphadenopathy and a diffuse ground glass process involving the right lower lobe suggestive of pneumonitis. A bronchoscopy was also performed. The bronchoalveolar lavage showed neutrophil predominant fluid, and cytology and adenosine deaminase were negative. Patient also had an endobronchial ultrasound-guided biopsy of the lymph nodes (EBUS). A work-up at this time was begun for drug induced vasculitis. She was treated empirically with steroids 40 mg for 10 days, and the methimazole was also discontinued. The antinuclear antibodies (ANA) came back positive with a speckled pattern; antineutrophil cytoplasmic antibody (c-ANCA) and antimyeloperoxidase MPO were also positive, but anti-double stranded DNA (DS-DNA) was negative. The clinical picture was in keeping with a drug-induced vasculitic picture which is unusual with the use of methimazole. The patient improved remarkably without recurrence of the pleural effusion. She was restarted on methimazole as it was not definite it was the cause of the pleural effusion. On a subsequent outpatient visit a couple of weeks after restarting the methimazole, the effusion recurred but did not require drainage. Methimazole was discontinued. She was referred for urgent thyroidectomy. Repeat chest X-ray showed complete resolution of the pleural effusion after stopping the methimazole. Repeat ANCA and antimyeloperoxidase antibody were both negative one and a half weeks after methimazole was discontinued.

### 2.1. Laboratory and Procedures

 See Table 1.

### 2.2. Imaging

 See Figures 1 and 2.

## 3. Discussion

This is the third published case of methimazole related pleural effusion [2, 3]. In our case, the unilateral pleural effusion was initially managed in the setting of atrial fibrillation with rapid ventricular rhythm. The consensus was that the dyspnea was from uncontrolled hyperthyroidism likely causing CHF exacerbation. However, even with optimization including rate control, thoracentesis, and antithyroid treatment, the patient was readmitted twice for a recurring unilateral pleural effusion. At the time of the second and third admission, the pleural effusion was exudative with neutrophilic predominance. The initial extensive work-up was largely negative, but, by the third admission, there was a suspicion that the effusion was related to the methimazole. She also tested positive for c-ANCA and MPO. Discontinuation of the methimazole with concomitant systemic steroids led to resolution of pleural effusion. Restarting the drug caused reaccumulation of the effusion, and withdrawing it caused its resolution without recurrence.

There are several notable differences among the three reported cases. For our case, the fluid was neutrophilic predominant. This may be explained in the context that the methimazole led to ANCA associated vasculitis leading to pleural effusion. This differs from the Gaspar-da-Costa et al.'s (BMC) case, which had eosinophilic exudative effusion with likely hypersensitivity immune drug reaction, and ANCA negative work-up [3]. The Sen et al.'s case showed a neutrophilic predominant effusion, but there is no mention of ANCA being tested. They had a histopathologic diagnosis of leukocytoclastic vasculitis; ANA was positive with a homogenous pattern but had negative anti-DS and anti-histone antibody [2]. Our patient had a positive ANA but with a speckled pattern without any other features concerning for lupus. Anti-DS DNA was negative also. These three cases show different profiles and possible mechanisms leading to the proposed methimazole-induced pleural effusion.

Thionamide side effects are uncommon and include fever, rash, arthralgias, agranulocytosis, and hepatitis as well as SLE like symptoms. Pleural manifestations associated with thionamides are relatively rare with only two cases associated with methimazole, two with PTU, and three with carbimazole. Propylthiouracil-induced vasculitis has been well described in several case reports, and most of them presented with pulmonary hemorrhage.

Doses of methimazole were not clearly mentioned in two case reports. Our patient was initially taking methimazole 20 mg daily prior to her first admission, but the dose was then increased to 30 mg daily during her first hospital stay. On review of other literature (Table 2), pleural effusion was reported starting from a dose of 10 mg of carbimazole.

Onset of pleural effusion may vary. It can be as short as 6 days, but it could also be long after thionamide therapy was started. The longest duration reported in the literature was 5 years after methimazole was initiated.

For control of our patient's hyperthyroid state, she received an urgent thyroidectomy due to concerns for worsening symptoms. Other thionamides were considered, but there was concern for recurrence of the pleural effusion as both PTU and Carbimazole have more reports of related pleural effusion. Radioactive iodine was considered, but the patient had a recent CT scan with contrast. In the ATS case, the patient also went for thyroidectomy. In the BMC case, the patient had type II amiodarone induced thyrotoxicosis, and no further treatment was needed after discontinuation of amiodarone. Detailed summary is provided in Table 2.

## 4. Conclusion

Our case report highlights the importance of the recognition of a rare side effect of methimazole. Timely diagnosis would ensure that appropriate treatment is given including corticosteroid administration, surgery, or radioiodine.

Table 2 is the summary of all the case reports of thioamide-induced pleural effusion.

## Figures and Tables

**Table 1:** Detailed laboratory and procedures.

Thyroid Function Tests	Initial Presentation: TSH <0.005 (0.358-3.740)
	Free T4 2.78 (0.76-1.46)
	Free T3 7.35 (2.18-3.98)
	9 days after medical optimization: TSH <0.005
	Free T4 1.54
	Free T3 2.80
	Hospital Course: TSH <0.005, Free T4 1.87, free T3 2.56

ANA	>1:80, positive and speckled pattern

Anti-Double stranded antibody	Negative

Antimyeloperoxidase antibody	Positive

c-ANCA	Positive→normal one and a half weeks after methimazole was discontinued.

p-ANCA	Negative

CT chest	Pleuritic, pneumonitis and mediastinal lymphadenopathy

CT abdomen	Ruled out intraabdominal etiologies such as malignancies

Bronchoscopy with EBUS	Negative for granulomatous disease, infection or malignancy

Bronchoalveolar Lavage (BAL)	Neutrophil predominant fluid with bacterial, fungal and AFB cultures negative.
	Cytology negative

Mediastinal Lymph Node Biopsy	Negative

**Table 2:** Summary of all the case reports of thioamide-induced pleural effusion.

Cases	Characteristics of pleural effusion	Thionamide	Treatment
Pedro Gaspar-da-Costa [3]	Eosinophils	Methimazole	Discontinued Methimazole

Gautam Das [5]	Exudative	Carbimazole	Switched to propylthiouracil (PTU)

Carol D Cardona Attard [6]	Exudative	Carbimazole	Switched to PTU and later total thyroidectomy

Ihteshamul Haq I [7]	Exudative	Carbimazole	Discontinued carbimazole and started prednisone

P. Sen [2]	Neutrophil predominant	Methimazole	Discontinued methimazole

Emmy YF Lau [4]	Pulmonary hemorrhage	Methimazole	Discontinued Methimazole and started prednisone

## References

[1] Thickett D. R., Richter A. G., Nathani N., Perkins G. D., Harper L. (2006). Pulmonary manifestations of anti-neutrophil cytoplasmic antibody (ANCA)-positive vasculitis. *Rheumatology*.

[2] Sen P., Dunn M. J. (2018). Its never lupus: a rare case of methimazole induced pleural effusion. *American Journal of Respiratory and Critical Care Medicine*.

[3] Gaspar-da-Costa P., Duarte Silva F., Henriques J. (2017). Methimazole associated eosinophilic pleural effusion: a case report. *BMC Pharmacology & Toxicology*.

[4] Lau E. Y. F., So S. Y., Chan E., Kwok J., Ma J., Kung A. W. C. (2009). Methimazole-induced antineutrophil cytoplasmic antibody-associated diffuse alveolar haemorrhage in a Chinese woman with Graves' disease. *Hong Kong Medical Journal*.

[5] Das G., Stanaway S. E. R. S., Brohan L. (2012). Carbimazole induced pleural effusion: a case report. *Case Reports in Endocrinology*.

[6] Attard C. D. C., Gruppetta M., Vassallo J., Vella S. (2016). Carbimazole-induced exudative pleural effusions. *BMJ Case Reports*.

[7] Haq I., Sosin M. D., Wharton S., Gupta A. (2013). Carbimazole-induced lupus. *BMJ Case Reports*.

